# Age at Diagnosis of Sickle Cell Anaemia in Lagos, Nigeria

**DOI:** 10.4084/MJHID.2013.001

**Published:** 2013-01-02

**Authors:** SO Akodu, IN Diaku-Akinwumi, OF Njokanma

**Affiliations:** Department of Paediatric, Lagos State University Teaching Hospital, Ikeja.

## Abstract

**Background:**

Sickle cell anaemia is the most common genetic disorder worldwide as well as in Nigeria. Delay in the diagnosis of the condition constitutes an important cause of concern for caretakers of affected children.

**Objective:**

To determine the age at diagnosis in a population of children with sickle cell anaemia in Lagos, Nigeria.

**Methodology:**

The study was conducted between October and December 2009 at the sickle cell clinic of the Department of Paediatrics of Lagos State University Teaching Hospital, Ikeja, Lagos in South west Nigeria. By convenience sampling, a total of 192 children with sickle cell anaemia aged six months to 15 years were interviewed with the aid of a structured questionnaire.

**Results:**

Overall, the mean age at confirmation of haemoglobin genotype was 27.33 months (± 26. 36 months). The mean age at diagnosis was significantly lower among males than females (25.59 ± 27.74 Vs. 29.14 ± 24.85, p = 0.04). A quarter of the children were diagnosed before infancy and three-quarters before three years of age. Upper social stratum and small family size were significantly associated with earlier diagnosis of sickle cell anaemia.

**Conclusion:**

Too few subjects are diagnosed in infancy. Routine screening should ideally be done at birth and neonatal period or at the latest, between six and nine months.

## Introduction

Sickle cell anaemia is one of the commonest single gene disorders in man with variable distribution in different parts of the world and variable clinical manifestations.^1^ Sickle cell anaemia commonly affects growth, leading to low mean weight, low mean height and decreased height velocity.^2^– ^6^

Africa has 70% of the world’s annual figure of 300,000 affected new births.^7^ In Nigeria, the prevalence of sickle cell trait is about 25% while the homozygous state is found in about 3% of the population.^8^ Nigeria has the largest population of people with sickle cell disorder, with about 150,000 births annually.^9^,^10^

Sickle haemoglobin is present at birth, but most infants don’t show signs until they are six months old or shortly before because the predominant haemoglobin at this time is foetal haemoglobin (Hb F).^8^ It has been earlier documented that high levels of Hb F inhibit sickling.^11^ This is because Hb F has the ability to decrease the polymerization of deoxygenated Hb S,^12^ hence preventing red blood cell from forming tactoids which lead to vaso-occlusion.^12^ Thus subjects with higher levels of persisting Hb F tend to have a milder disease course and better prognosis. Also, a higher concentration of Hb F implies lower concentrations of Hb S within the cell and hence lower likelihood of untoward manifestations and complications.^13^–^16^

Sickle cell anaemia accounted for 8.2% of all admissions and 24.6% of those who had severe anaemia in a study at the children’s emergency ward, University College Hospital, Ibadan.^17^ This confirmed that sickle cell anaemia is an important cause of severe anaemia and of hospital admissions.

Despite having the highest burden of sickle cell disease in the world,^9^,^10^ Nigeria does not have a neonatal screening program and in the majority of cases the diagnosis is made when medical care is sought for symptoms. Early diagnosis of sickle cell anaemia allows prevention of complications and prompt treatment,^18^–^20^ thus reducing morbidity and mortality.^21^

Lagos is a cosmopolitan State, and the largest and the most populous urban Center in Nigeria.^22^ Being the commercial nerve Center of the country, it attracts different ethnic groups, and this makes it very heterogeneous and suitable for population studies. At present, the mean age at diagnosis of sickle cell anaemia in a megacity like Lagos is unknown. The main objective of this study was to describe the age at diagnosis in children with sickle cell anaemia in Lagos, Nigeria in those who were not subjected to prenatal/neonatal screening. It is hoped that this study will stimulate a more purposeful awareness campaign about genetic counselling, prenatal diagnosis and neonatal screening on sickle cell anaemia in Nigeria.

## Methodology

The study is a descriptive cross-sectional facility-based one conducted between October and December 2009 among children with sickle cell anaemia attending the sickle cell clinic of the Department of Paediatrics of Lagos State University Teaching Hospital, Ikeja, Lagos in South west Nigeria. The Lagos State University Teaching Hospital (LASUTH) is an urban tertiary health centre and a major referral center for the whole region.

Approval for the study was obtained from the Ethics Committee of LASUTH. Consecutive sickle cell anaemia patients who came for routine follow up clinic were recruited. One hundred and ninety-two children with haemoglobin genotype SS were studied. The haemoglobin genotype was determined using cellulose acetate paper in alkaline electrophoresis combined with sickling test.

Data collection was with the aid of pre-tested structured questionnaires conducted by a single investigator. The questionnaire was pre-tested on a small number of similar respondents to identify the likely problems and to eliminate them. Questions covered demographic details with age at diagnosis as well as circumstances surrounding diagnosis. Social classification was done using the scheme proposed by Oyedeji^23^ which relies on parental educational level and occupation. The system defines five socioeconomic classes, I to V, in descending order of privilege. Classes I and II were grouped together as upper social stratum while classes IV and V were grouped together as lower social strata; class III was considered middle stratum. The data was analyzed using Statistical Package for Social Science (SPSS). Tests of statistical significant were by Mann-Whitney-U test and level of significance set at p < 0.05.

## Results

### Characteristics of the study population

A total of 192 children with haemoglobin genotype SS whose caregiver consented to participate in the study were recruited over a study period of three months (October 2009 through December 2009). The age and gender distributions of the study patients are given in Table 1. Overall, the age of the subjects ranged from nine months to 15 years, with a mean of 55.09 (±43.57) months and a median of 42.00 months.

### Age at diagnosis

The age at which the subjects had their haemoglobin genotype status confirmed range from three months to 12 years, with a mean age of 27.33 (±26.36) months and a median of 18 months. The modal age category at which the subjects had their haemoglobin genotype status confirmed was 1 – <3 years age group (table 2). The cumulative frequency is used to determine the number of observations that lie above (or below) a particular value in a data set. A quarter of the study subjects had their haemoglobin genotype status confirmed before the age of one year. One infant was diagnosed in the United Kingdom prior to enrollment at LASUTH before the age of six months. Almost three-quarter of the subjects were diagnosed before three years of age.

Overall, the age at confirmation of haemoglobin genotype was significantly higher among female respondents than male respondents (p = 0.04). This pattern was observed in all age-groups except for those below five years (table 3). On the contrary however, there was no significant difference when the results were analyzed according to age-groups (p>0.05).

Analysis of family setting and age at diagnosis of sickle cell anaemia among the respondents is shown in Table 4. Diagnosis was made earlier among children whose parents were married, those from monogamous homes, with family size less than five and those of second or higher birth order. However, the observed differences were not significant (p > 0.05). The mean age at diagnosis was significantly lower among children of upper socioeconomic status (p = 0.03).

## Discussion

In the region of Lagos, neither prenatal nor neonatal screening for sickle cell anaemia is offered. So the age at which children are diagnosed with sickle cell anaemia has been unknown so far. Also, haemoglobin genotype determination is not performed on a routine basis; it is only done if requested by child’s caregivers or healthcare providers.

Early diagnosis of sickle cell anaemia is very important because many complications can be prevented by early diagnosis and treatment as well as by education of the parents on complications requiring immediate care. Newborn screening for sickle cell disease in California, USA, has shown that screening, coupled with extensive follow-up, reduces mortality.^19^, ^20^ Improved survival of screened babies has also been confirmed by the Jamaican cohort study that showed a reduction of early causes of death such as acute splenic sequestration, pneumococcal septicemia, aplastic crisis, and acute chest syndromes.^24^

The age at which diagnosis of sickle cell anaemia was made had a wide range and standard deviation for two reasons – absence of a routine screening programme for the diagnosis and the fact that there is no specific age at which manifestations attract attention of parents/guardians. Nearly three quarters of our subjects were diagnosed before the age of three while more than 10% were diagnosed after their fifth birthday. It is plausible that a number of factors, particularly social and health circumstances could have contributed to earlier diagnosis of some of our children. In the absence of a routine screening program, diagnosis is often made when patients show up with suggestive clinical features. Early diagnosis may in turn depend on health-seeking attitude of caregivers among other factors. In any case, the establishment of a routine screening programme which could be prenatal, neonatal or tied to child welfare services like immunization would significantly reduce the number of children with delayed diagnosis for any reason. The major limitation of our report is that we do not know the circumstances under which the diagnosis was made in individual subjects. If for instance, diagnoses were mostly made because the children presented with suggestive history or physical findings, differences in type and severity of initial presenting features would explain differences in age at diagnosis. If on the other hand, the parents requested the tests, health–seeking behaviour would be called to question. If the children had visited hospital and/or had blood tests a number of times before the diagnosis, lack of a surveillance system that would demand routine haemoglobin genotype screening would be at fault.

The present study showed that the overall mean age at diagnosis of study subjects was significantly higher among female subjects than male subjects. The explanation for the different pattern in females and males may be because male is more prone to sickle cell crisis as they are more exposed to known precipitating factors.^25^ Lack of significant difference emerged on stratification according to age-groups. It is possible that the lower numbers attendant upon such stratification was responsible for the loss of significance.

The study also revealed that an upper socioeconomic stratum was associated with younger age at diagnosis of sickle cell anaemia. This finding is consistent with that of Brown et al^26^ in Ibadan. It is logic to argue that upper socioeconomic stratum implies that there is better access to health care by such families as well as privilege to health information.

It is straightforward that a routine screening at some point is imperative in Lagos. This may be done prenatally, in the neonatal period or sometime in the first year of life using DNA analysis or Agar gel electrophoresis respectively. Some workers^27^ have reported screening in the first year of life through infant welfare clinics and well baby clinics e.g. during the measles immunization visit at nine months as a viable approach in developing countries with limited resources.

## Figures and Tables

**Table 1:** Distribution of study subjects.

Age group (years)	Male	Female	Total
0 – <1	4 (50.0)	4 (50.0)	8
1 – <3	41 (53.9)	35 (46.1)	76
3 – <5	23 (46.9)	26 (53.1)	49
5 – <10	17 (53.1)	15 (46.9)	32
10 – 15	13 (48.1)	14 (51.9)	27

Total	98 (51.0)	94 (49.0)	192

NB: Values in parenthesis are % of rows total

**Table 2:** Distribution of study subjects according to age group at diagnosis.

Age group at diagnosis (years)	Frequency	Percent	Cumulative percent
0 – <1	49	25.5	25.5
1 – <3	93	48.4	73.9
3 – <5	30	15.6	89.5
5 – <10	16	8.3	97.8
10 – 15	4	2.2	100.0

**Table 3:** Age at diagnosis according to gender.

Age group (years)	Age at Diagnosis (months)			
	Male	Female	MWU-value	p-value
0 – <1				
Mean (SD)	8.75 (2.06)	8.25 (2.22)	7.00	0.77
Median	9.00	8.00		
1 – <3				
Mean (SD)	18.10 (21.61)	16.31 (11.57)	714.00	0.97
Median	14.00	15.00		
3 – <5				
Mean (SD)	26.22 (29.39)	24.54 (12.87)	250.00	0.32
Median	12.00	24.00		
5 – <10				
Mean (SD)	30.18 (23.11)	37.13 (16.35)	95.00	0.22
Median	24.00	36.00		
10 – 15				
Mean (SD)	45.25 (40.27)	67.14 (34.80)	58.00	0.11
Median	34.50	69.00		
Total				
Mean (SD)	25.59 (27.74)	29.14 (24.85)	3810.00	0.04
Median	14.50	21.50		

Family setting, socioeconomic status and age at diagnosis

**Table 4:** Age at haemoglobin genotype confirmation according to family setting.

	Frequency	Mean age at diagnosis (months)	p-value
	n (%)	Mean (SD)	
**Marital status of parents**			
Married	185 (96.40)	27.15 (26.45)	0,47
Others	7 (3.60)	32.14 (25.26)	
**Family structure**			
Monogamous*	173 (93.50)	26.29 (26.15)	0,11
polygamous*	12 (6.50)	39.50 (28.84)	
**Family size**			
<5	81 (42.18)	23.22 (23.25)	0,08
≥5	111 (57.82)	30.32 (28.13)	
**Birth order of subjects**			
1	65 (33.90)	29.48 (29.05)	0,72
≥2	127 (66.10)	26.23 (24.92)	
**Socioeconomic strata**			
Upper strata	77 (40.10)	22.66 (25.06)	0,03
Others	115 (59.90)	30.45 (26.85)	

* Based on the 185 settings in which parents were married
